# Temporal and spatial trends in road traffic fatalities from 2001 to 2019 in Shandong Province, China

**DOI:** 10.1371/journal.pone.0287988

**Published:** 2023-07-07

**Authors:** Tao Wang, Zhi-Ying Yao, Bao-Peng Liu, Cun-Xian Jia

**Affiliations:** Department of Epidemiology, School of Public Health, Cheeloo College of Medicine, Shandong University, Jinan, Shandong, China; Southeast University, CHINA

## Abstract

**Objective:**

This study explored the temporal and spatial trends in road traffic fatalities in Shandong Province from 2001 to 2019 and discusses the possible influencing factors.

**Methods:**

We collected data from the statistical yearbooks of the China National Bureau of Statistics and the Shandong Provincial Bureau of Statistics. Join-point Regression Program 4.9.0.0 and ArcGIS 10.8 software were used to analyze the temporal and spatial trends.

**Results:**

The mortality rate of road traffic injuries in Shandong Province decreased from 2001 to 2019, with an average annual decrease of 5.8% (*Z* = −20.7, *P* < 0.1). The three key time points analyzed in the Join-point regression model roughly corresponded to the implementation times of traffic laws and regulations in China. The temporal trend in case fatality rate in Shandong Province from 2001 to 2019 was not statistically significant (*Z* = 2.8, *P* < 0.1). The mortality rate showed spatial autocorrelation (global Moran’s *I* = 0.3889, *Z* = 2.2043, *P* = 0.028) and spatial clustering. No spatial autocorrelation was observed in the case fatality rate (global Moran’s *I* = −0.0183, *Z* = 0.2308, *P* = 0.817).

**Conclusions:**

The mortality rate in Shandong Province decreased significantly over the studied period, but the case fatality rate did not decline significantly and remains relatively high. Many factors influence road traffic fatalities, among which laws and regulations are the most important.

## 1. Introduction

Road traffic injuries (RTIs) are a global public health problem, and death is the worst outcome of an RTI. The *Global Status Report on Road Safety 2018* issued by the World Health Organization (WHO) [1] indicates that RTIs are the eighth most prominent cause of death among all people and the leading cause of death for people aged 5–29. RTIs are a serious problem that cannot be ignored. While the total number of vehicles in low- and middle-income countries accounts for only 60% of the world’s total, they make up 93% of the road traffic fatalities [2]. In China, RTIs are the most common cause of injury-related death, with a mortality rate of 14.23 per 100,000 [3]. Consequently, RTIs seriously threaten health and impose a heavy disease burden on society, families, and individuals.

Due to the serious consequences of RTIs, the United Nations and WHO have issued related guidelines, and the incidence and mortality rate of road traffic accidents have gradually decreased in some countries [4–6]. However, these metrics continue to rise in middle—and low-income countries [7]. Since 2004, China has successively introduced mandatory laws and regulations to improve road safety, including the “Road Traffic Safety Law of the People’s Republic of China” (2004), severe punishments for alcohol-impaired driving (2009), and sentences for driving after drinking (2011). Although the overall mortality rate of traffic accidents in China is decreasing, its burden remains severe [8–10]. RTIs in China are mainly distributed in economically developed provinces such as Zhejiang and Jiangsu Province [11]. These areas have a high level of private car ownership and heavy road traffic, resulting in frequent RTIs.

China’s economy is in a period of rapid development, and road construction and the number of vehicles are also growing rapidly. Although vehicles provides users with convenient transportation, they also bring security risks. As the number of motor vehicles increases, traffic accidents will also increase. In addition to traditional modes of transportation, new modes such as electric bicycles, shared bicycles, and shared cars are also widely used in China, and RTIs involving these modes are increasing year by year [12, 13]. For example, a study in Zhejiang Province in China found that accidents caused by e-bikes accounted for one third of all traffic accidents from 2008 to 2011 [14]. However, even with the continued emergence of alternative transportation modes, motor vehicles remain the leading mode in road traffic fatalities [15]. In addition to traffic collisions caused by vehicles, imperfect road safety measures can also lead to traffic accidents. The Haddon model [16] indicates that road disrepair and infrastructural inadequacy are among the factors contributing to traffic collisions. Thus, road traffic safety is threatened by an increasing number of vehicles and a lack of road safety measures, which is compounded by the proliferation of new transportation modes. Prior thinking considered RTIs to be accidental and inevitable. However, given the inherent regularity in the occurrence of RTIs, they can be prevented [17]. Exploring the factors influencing RTIs and intervening accordingly are essential to improving road safety.

Traffic flow [18–20] and road density [21, 22] are factors that can be used to predict traffic accidents. Shandong Province is located in the core area of North–South transportation and has a well-developed road network. China’s statistical yearbook for 2019 [23] shows that the total road mileage in Shandong Province has reached 280,300 km, ranking third in China. At the same time, it has better economic strength in this province. The traffic flow is heavy on both the expressway and the arterial road. In addition, Shandong Province is characterized by its large area and population. A study has shown that the higher the population density, the more likely traffic injuries are to occur, especially serious traffic injury [22]. Therefore, the risk of road traffic injuries and even fatalities in Shandong Province is extremely high. This study has practical significance for preventing road traffic fatalities in Shandong Province.

In this study, we used Join-point regression and spatial autocorrelation models to analyze the temporal and spatial trends in road traffic fatalities in Shandong Province and identify the key nodes. We also investigated the key factors influencing road traffic fatalities in Shandong Province to inform the development of targeted interventions to reduce preventable fatalities.

## 2. Data sources and methods

### 2.1 Data sources

The datasets generated for this study can be found in the China National Bureau of Statistics and the Shandong Provincial Bureau of Statistics [23, 24]. All of the road traffic fatalities data is from the Bureau of Statistics at https://data.stats.gov.cn/and http://tjj.shandong.gov.cn/. The data included: the number of people who died in traffic accidents; the number of people injured in traffic accidents; the total population at the end of the year; and road network characteristics (length of highways, second class and above, length of expressway and road density).

### 2.2 Data management and statistical analysis

Missing data were interpolated using the linear trend of adjacent points in IBM SPSS Statistics 24.0 software.

The authors calculated mortality and case fatality rate as indexes to reflect the seriousness of road traffic accidents.


$$Mortality\;rate=\frac{k}{p}*\frac{100.000}{100,000}$$ (1)

$$Case\;fatality\;rate=\frac{k}{n}*100\%$$ (2)

Where k is the number of road traffic fatalities in Shandong Province in a period of time; p is the total population of Shandong Province during the same period; n is the number of road traffic accidents in Shandong Province during the same period.

The mortality and case fatality rate of road traffic accidents are influenced by various factors. Therefore, a multiple linear regression model was formulated with the mortality and case fatality rate as dependent variables, respectively, and the calculated formula was as follows:

$$y_{i}=\beta_{0}+\sum_{k=1}^{k}x_{ki}\beta_{k}+u_{i}$$ (3)

where *β*_0_ is the intercept, *i* = 1,2,……n, k is the number of explanatory variables. *u*_*i*_ is the random error. *β*_*k*_ is the partial regression coefficient, which indicates the change in the mean of the dependent variable for each unit change in *x*_*k*_, with the other explanatory variables held constant.

The temporal trend was analyzed by segmented regression analysis using Join-point regression 4.9.0.0 software developed by the National Cancer Institute. Three time points were connected in the Join-point regression analysis. The annual percentage change (APC) and average annual percentage change (AAPC) with corresponding 95% confidence intervals (*95%CI*) were calculated to determine if the changes in the overall trend and the trends in each segment were statistically significant.

APC and AAPC are calculated based on the following formula:

$$In(y|x)=b_{0}+b_{1x}$$ (4)

$${APC=100*(exp}^{b_{1}}-1)$$ (5)

$$AAPC=\left\{exp(\frac{\sum w_{i}b_{i}}{\sum w_{i}})-1\right\}*100$$ (6)

where x is the year, y is the mortality or case fatality rate, *b*_0_ is the intercept, *b*_*i*_ is the regression coefficient, which is the slope coefficient of each line segment, and *w*_*i*_ is the number of years included in each time segment.

ArcGIS 10.8 software was used to associate city codes with the road traffic fatality database, establish the geographic information database, and construct the spatial distribution map of mortality rate and case fatality rate from 2011 to 2019. The geographical clustering of road traffic deaths was conducted using global spatial autocorrelation and local spatial autocorrelation.

Moran’s *I* is used to indicate the global spatial autocorrelation, which is calculated as follows:

$$I=\frac{n*\sum_{i=1}^{n}\sum_{j=1}^{n}w_{ij}(x_{i}-\bar{x})(x_{j}-\bar{x})}{(\sum_{i=1}^{n}\sum_{j=1}^{n}w_{ij})*\sum_{i=1}^{n}{\left(x_{i}-\bar{x}\right)}^{2}},i\neq j$$ (7)

Local Moran’s *I* is used to describe the local spatial autocorrelation, which is calculated as follows:

$$I_{i}=\frac{n(x_{i}-\bar{x})\sum_{j}w_{ij}(x_{j}-\bar{x})}{{\sum_{i}\left(x_{i}-\bar{x}\right)}^{2}}$$ (8)

In this study, n means the number of cities with road traffic fatalities; *x*_*i*_ means the road traffic injury mortality and case fatality rate of the city; $\bar{x}$ is the average of road traffic injury mortality and case fatality rate of all cities in Shandong Province, and W_*ij*_ is the spatial weight coefficient of the *i*-th and *j*-th regions.

All tests were two-sided, with *P* < 0.05 considered statistically significant.

## 3. Results

### 3.1 Basic information on road traffic fatalities

From 2001 to 2019, the total number of fatalities from road traffic accidents in Shandong Province was 101,493, the average mortality rate was 5.58/100,000, and the average case fatality rate was 24.94%. The annual mortality rate for road traffic accidents decreased from 10.33/100,000 in 2001 to 3.51/100,000 in 2019. From 2001 to 2011, the case fatality rate increased, peaked in 2011 (29.71%), and then declined (Table 1).

**Table 1:** Basic information on road traffic accidents in Shandong Province from 2001 to 2019.

Year	Population (100, 000)	Number of traffic accidents	Number of deaths	mortality rate (/100, 000)	case fatality rate (%)
2001	904.1	43698.3	9339	10.33	21.37
2002	908.2	56818	9167	10.09	16.13
2003	912.5	49414	8905	9.76	18.02
2004	918.0	39815	7804	8.50	19.60
2005	924.8	35251	7050	7.62	20.00
2006	930.9	30056	6309	6.78	20.99
2007	936.7	25867	5760	6.15	22.27
2008	941.7	19594	5026	5.34	25.65
2009	947.0	16166	4518	4.77	27.95
2010	958.8	14560	4268	4.45	29.31
2011	966.5	13375	3974	4.11	29.71
2012	970.8	13275	3838	3.95	28.91
2013	974.6	12879	3748	3.85	29.10
2014	980.8	13570	3703	3.78	27.29
2015	986.6	13376	3652	3.70	27.30
2016	997.3	13163	3614	3.62	27.46
2017	1003.3	13403	3665	3.65	27.34
2018	1007.7	13226	3600	3.57	27.22
2019	1010.6	13150	3553	3.51	27.02
Total	-	-	-	5.58	24.94

### 3.2 The relationship between road network characteristics and the severity of road traffic accidents

The results of multiple linear regression analysis (Table 2) showed that road density was an influential factor in mortality rate with a regression coefficient of -0.012, indicating that mortality rate would decrease as road density increased. The length of highways, length of expressways, and road density were the factors influencing the case fatality rate. The regression coefficient of length of highways was 0.001, indicating that as the length of highways increases, the case fatality rate will increase. The regression coefficients of length of expressways and road density were -0.042 and -0.151, respectively, indicating that the case fatality rate would decrease as the length of expressways and road density increased.

**Table 2:** The relationship between road network characteristics and the severity of traffic accidents.

	mortality rate				case fatality rate			
	*β*	SE	t	*P-*value	*β*	SE	t	*P-*value
Interpret	6.060	0.691	8.771	<0.001	63.837	8.962	7.123	<0.001
Length of highways (km)	0.000	0.000	-0.231	0.818	0.001	0.000	4.384	<0.001
Second class and above (km)	0.000	0.000	-0.769	0.443	-0.002	0.002	-1.231	0.220
Length of expressway (km)	0.001	0.001	1.190	0.236	-0.042	0.009	-4.418	<0.001
Road density (km/100 sq.km)	-0.012	0.004	-3.003	0.003	-0.151	0.053	-2.870	0.005

### 3.3 Join-point regression analysis of the severity of road traffic accidents

**Fig 1:**
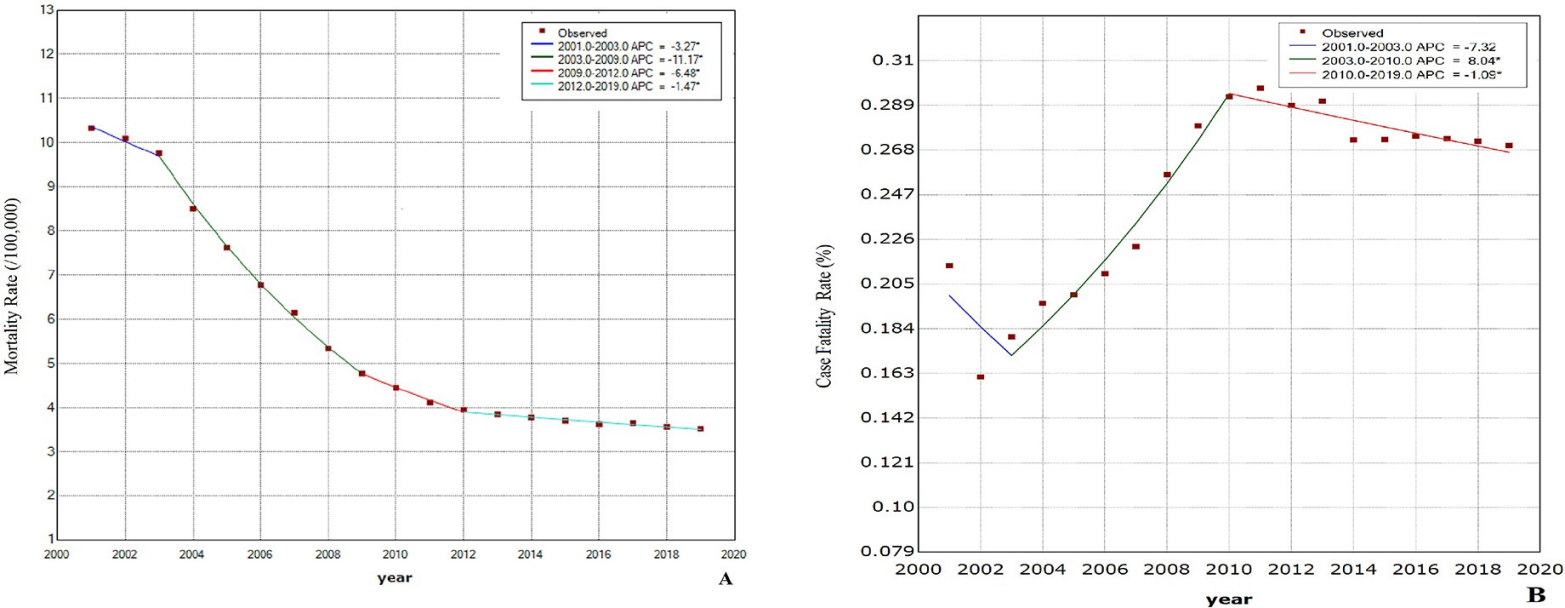
Fitting trend chart of Join-point regression model for mortality rate and case fatality rate in Shandong Province from 2001 to 2019. (A: mortality rate, B: case fatality rate).

The Join-point regression model was used to analyze the temporal trends in mortality rate and case fatality rate (Table 3 and Fig 1). Overall, the mortality rate decreased from 2001 to 2019, with an average annual decline of 5.8% (*P* < 0.1). The decline was most evident from 2003–2009 (APC = −11.2%, *P* < 0.001) followed by 2009–2012 (APC = −6.5%, *P* = 0.001), while the decline was slowest from 2012–2019 (APC = −1.5%, *P* < 0.001). The case fatality rate of road traffic accidents from 2001 to 2019 increased by an average of 1.6% per year (*P <* 0.1); an upward trend was observed from 2003–2010 (APC = 8.0%, *P* < 0.001), while a downward trend was observed from 2010–2019 (APC = −1.1%, *P* = 0.015). The trend from 2001–2003 was not statistically significant (APC = −7.3%, *P* = 0.095).

**Table 3:** Join-point regression analysis of the severity of road traffic accidents in Shandong Province from 2001 to 2019.

		mortality rate (/100, 000)	case fatality rate (%)
Segmentation 1	Period	2001–2003	2001–2003
	APC (*95%CI*)	-3.3 (-6.2, -0.3)	-7.3 (-15.4, 1.6)
	*t*	-2.5	-1.8
	*P*-value	0.036	0.095
Segmentation 2	Period	2003–2009	2003–2010
	APC (*95%CI*)	-11.2 (-11.80, -10.6)	8.0 (6.4, 9.7)
	*t*	-40.1	11.0
	*P*-value	< 0.001	< 0.001
Segmentation 3	Period	2009–2012	2010–2019
	APC (*95%CI*)	-6.5 (-9.3, -3.6)	-1.1 (-1.9, -0.3)
	*t*	-5.1	-2.9
	*P*-value	0.001	0.015
Segmentation 4	Period	2012–2019	-
	APC (*95%CI*)	-1.5 (-1.9, -1.1)	-
	*t*	-8.4	-
	*P*-value	< 0.001	-
AAPC (*95%CI*)		-5.8 (-6.4, -5.3)	1.60 (0.5, 2.8)
Z		-20.7	2.8
*P*-value		< 0.1	< 0.1

### 3.4 Spatial distribution of the mortality rate from road traffic accidents

The analysis of spatial distribution revealed that the mortality rates in all cities decreased over the study period. In 2011, the mortality rate in Shandong Province was 4.12/100,000. The highest mortality rates were found in Dongying (8.56/100,000), Binzhou (7.96/100,000), Zibo (7.57/100,000), and Weihai (6.43/100,000). Linyi (1.28/100,000) had the lowest mortality rates. The total mortality rate in 2015 was 3.70/100,000, with the highest rates found in Zibo (6.81/100,000), Dongying (6.11/100,000), Jinan (5.89/100,000), and Weihai (5.10/100,000). The mortality rate was lowest in Heze (1.80/100,000). In 2019, the overall mortality rate was 3.53/100,000, with the highest rates found in Zibo (6.73 /100,000) followed by Dongying (5.87/100,000), Jinan (5.30/100,000), and Weihai (4.94 /100,000). Heze (1.57/100,000) had the lowest rate.

### 3.5 Spatial distribution of case fatality rate from road traffic accident

The analysis of spatial distribution revealed that the case fatality rate in all cities decreased over the study period. In 2011, the case fatality rate in Shandong Province was 29.71% (3974/13375), with the highest rates found in Heze (173/193, 89.64%) followed by Weihai (180/223, 77.25%), Binzhou (300/569, 52.72%), Tai’an (234/529, 44.23%), and Zaozhuang (134/329, 40.73%). Qingdao (374/2164, 17.28%) had the lowest fatality rate. In 2015, the case fatality rate in Shandong Province was 27.21% (3652/13376), with the highest rates found in Heze (153/177, 86.44%), Weihai (143/172, 83.14%), Linyi (274/497, 55.13%), Binzhou (113/262, 43.13%), and Tai’an (189/466, 40.56%). Qingdao (313/1814, 17.25%) and Jinan (500/3232, 15.47%) had the lowest case fatality rate in 2015. The case fatality rate in 2019 was 27.05%, with the highest rates found in Heze (138/156, 88.46%), Weihai (140/170, 82.35%), Linyi (288/522, 55.17%), Binzhou (112/273, 41.03%), and Tai’an (193/475, 40.63%). Jinan (472/3334, 14.16%) had the lowest case fatality rate in 2019.

### 3.6 Spatial autocorrelation analysis of mortality rate and case fatality rate for road traffic accidents from 2011 to 2019

Based on the global spatial autocorrelation analysis of mortality rate, the global Moran’s *Ⅰ* index was 0.3889 with *Z* = 2.2043 and *P* = 0.028. Thus, the mortality rate showed a spatial correlation. The results of further local spatial autocorrelation analysis showed that Binzhou was found to be a high–high cluster for mortality rate (i.e., the mortality rate was high in Binzhou and in its neighboring cities). Rizhao emerged as a high–low cluster, meaning that the mortality rate was high in Rizhao but low in the surrounding cities. Zaozhuang was a low–low cluster, with a relatively low mortality in both the city and surrounding cities. The mortality rate in other cities had no spatial correlation. The global spatial autocorrelation analysis of the case fatality rate indicated a global Moran’s *Ⅰ* index was −0.0183 with *Z* = 0.2308 and *P* = 0.817. No spatial correlation was found in case fatality rate.

## 4. Discussion

We analyzed the temporal and spatial trends in the mortality rate and case fatality rate for road traffic accidents in Shandong Province. Overall, the mortality rate in Shandong Province has shown a downward trend over the past 20 years, while the case fatality rate increased rapidly and then gradually declined from 2001 to 2019. The mortality rate also showed spatial clustering. We also discuss the possible influencing factors to provide a theoretical basis for preventing road traffic fatalities through the temporal and spatial trend analysis of road traffic fatalities.

Over the past 20 years, the mortality rate of road traffic accidents has declined worldwide [6]. Nevertheless, upward trends persisted in some countries and regions [25]. Road traffic laws and regulations are decisive factors influencing road traffic fatalities. In 2018, South Korea enacted new drunk-driving laws, and alcohol-related road traffic death rates decreased by more than 15.0% [26]. Under new alcohol control policy measures, alcohol-related traffic injury in Lithuania decreased markedly [27]. In addition to laws and regulations, economic development is another influential factor. As the economy develops, the road traffic mortality rate usually decreases [28]. According to a study, the road traffic mortality rate in China was 21.8% lower in 2017 than in 1990 [29]. We obtained similar results in this study. Although the road traffic mortality rate in Shandong Province is declining, the disease burden remains significant. RTIs are the leading cause of death among people aged 15–64 and the leading cause of premature death in the population [30].

The time points used to analyze the temporal trend in road traffic mortality rate via Join-point regression are consistent with the implementation times of road traffic-related laws and regulations in China. The severe legal consequences imposed by these laws and regulations help restrain dangerous driving behavior. Implementing such laws and regulations can provide a safer traffic environment for all people and especially vulnerable road users, thereby reducing road traffic accidents and fatalities.

Drivers are the main initiators of road traffic accidents. A driver’s lack of sufficient driving knowledge and skills can lead to careless driving behavior, leading to road traffic accidents [16]. Driver education can affect drivers’ understanding of traffic signs and regulations. Therefore, improving the education of drivers can reduce road traffic accidents [31]. With the popularization of the compulsory education systems in China and the gradual expansion of higher education, education is no longer just for the elite, and the average level of education has steadily increased. After the reform and opening up, the educational level of Shandong Province improved significantly in both scale and quality [32]. Improving the education level can help reduce road traffic fatalities in this province.

In addition to laws and regulations, improving the accuracy of weather forecasts, deploying speed-measuring radar, and the Skynet project can also help reduce road traffic accidents. While China has implemented various measures to prevent road traffic accidents with remarkable effect, some shortcomings remain. For example, there are no mandatory requirements for child safety seats.

Economic development improves quality of life and gradually increases motor vehicle ownership. Shandong Province has one of the highest levels of car ownership in China. Motor vehicle ownership is positively related to traffic accidents; for every 1% increase in motor vehicle ownership, the number of traffic accidents increases by 2.83% [33]. The increased motor vehicle ownership brought about by economic development is inevitable. Therefore, it is necessary to strengthen vehicle safety devices to lessen the severity of vehicle collisions and improve the safety of drivers and passengers. The newly revised national standards for the safe operation of motor vehicles reinforce the equipment and structural safety requirements for motor vehicle safety devices.

Investment is another factor affecting traffic safety [34]. Investment includes two aspects: the construction of transportation facilities and road maintenance. Potholes or fading of the road surface caused by inadequate road maintenance contribute to road traffic accidents [35]. In the past 20 years, relevant organizations in Shandong Province have continuously explored new road maintenance models [36]. Establishing a scientific and practical road maintenance management system can provide a good road environment and reduce road traffic accidents.

Medical treatment is another factor influencing the fatality rate [37]. The timely treatment of injuries can decrease the probability of death and disability in traffic accidents. In 2010, China established a more extensive and inclusive rural cooperative medical insurance system covering urban and rural residents. Shandong Province achieved the full provincial implementation of this Medicare system in 2009, earlier than its nationwide implementation. With the comprehensive implementation and constant improvement of the medical insurance system, the influx of medical insurance funds has increased. In addition, the numbers of medical staff and institutions have also increased year by year [38]. Timely care can reduce deaths caused by a lack of access to medical treatment and money.

In this study, we found non-random spatial clustering in the road traffic mortality rate in Shandong Province. One factor affecting the clustering is differences in economic development. Although both high–high and low–low clusters were found among poor areas in Shandong Province, the high–high cluster area has abundant mineral resources and dominant secondary industry, resulting in numerous heavy and oversized vehicles and a large freight volume. In contrast, the low–low cluster areas are dominated by primary and tertiary industries [39]. Primary and secondary industries are positively correlated with road traffic accidents, while the opposite relationship is observed for tertiary industry [40].

This study found that the unevenness of road network characteristics is another factor contributing to spatial clustering. According to the statistical yearbook of Shandong Province, the road density in the high–high cluster area is lower than in the low–low cluster area. The increase in road density improves the efficiency of traffic circulation and optimizes traffic operations for better accessibility [41–43]. This weakens the probability and severity of traffic accidents and reduces the mortality rate of accidents. The driver’s concentration level while driving can also affect road traffic accidents (e.g., through distracting driving) [44]. Drivers are more focused on driving in mountainous areas than in flatlands. In the high–high cluster areas of Shandong Province, publicity and education on road safety and increased road safety inspections by law enforcement can help reduce road traffic fatalities.

This study is ecological, and the results may have ecological fallacies. Using individual data can help validate the results of this study. We did not analyze data on road traffic fatalities in the smaller administrative regions because the Bureau of Statistics for some cities does not make the relevant data publicly available.

## 5. Conclusion

We analyzed the temporal and spatial trends in road traffic fatalities based on data from the China National Bureau of Statistics and the Shandong Provincial Bureau of Statistics. The mortality rate in Shandong Province has shown a downward trend over the past 20 years, and spatial clustering in mortality rate was observed. The case fatality rate first increased rapidly and then slowly decreased from 2001 to 2019, and no spatial autocorrelation was found. Road traffic laws and regulations are the most important factors influencing traffic safety. This study provides a theoretical basis for the government to formulate measures to reduce road traffic fatalities and protect human life.
